# Synthesis, Molecular and Crystal Structure Analysis of 1-(4-Methylbenzenesulfonyl)indole-3-carbaldehyde and DFT Investigation of Its Rotational Conformers

**DOI:** 10.3390/molecules19021990

**Published:** 2014-02-13

**Authors:** Julio Zukerman-Schpector, Lucas Sousa Madureira, Glaudeston Dutra Wulf, Hélio A. Stefani, Stanley N. S. Vasconcelos, Seik Weng Ng, Edward R. T. Tiekink

**Affiliations:** 1 Laboratório de Cristalografia, Estereodinâmica e Modelagem Molecular, Departamento de Química, Universidade Federal de São Carlos, C.P.676, São Carlos, SP, 13565-905, Brazil; E-Mails: lucassousamadureira@gmail.com (L.S.M.); glaudeston_@hotmail.com (G.D.W.); 2 Departamento de Farmácia, Faculdade de Ciências Farmacêuticas, Universidade de São Paulo, 05508-900, SP, Brazil; E-Mails: hstefani@usp.br (H.A.S.); stanleynsv@gmail.com (S.N.S.V.); 3 Department of Chemistry, The University of Malaya, Kuala Lumpur 50603, Malaysia; E-Mail: mseikweng@um.edu.my; 4 Chemistry Department, Faculty of Science, King Abdulaziz University, Jeddah 80203, Saudi Arabia

**Keywords:** indole-3-carbaldehyde, conformational isomerism, DFT, IRC, crystal structure analysis, X-ray diffraction

## Abstract

Two independent molecules that differ in terms of rotation about the central S-N bond comprise the asymmetric unit of the title compound **1**. The molecules have a V-shape with the dihedral angles between the fused ring system and benzene ring being 79.08(6)° and 72.83(5)°, respectively. The packing is mostly driven by π···π interactions occurring between the tolyl ring of one molecule and the C_6_ ring of the indole fused ring system of the other. DFT and IRC calculations for these and related 1-(arylsulfonyl)indole molecules showed that the rotational barrier about the S-N bond between conformers is within the 2.5–5.5 kcal/mol range. Crystal data for C_16_H_13_NO_3_S (**1**): *M*r = 299.33, space group *Pna*2_1_, *a* = 19.6152(4) Å, *b* = 11.2736(4) Å, *c* = 12.6334(3) Å, *V* = 2793.67(13) Å^3^, *Z* = 8, *Z*' = 2, *R* = 0.034.

## 1. Introduction

**Figure 1:**
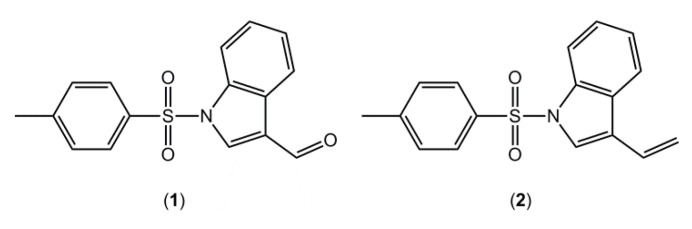
Chemical structures of (**1**) and (**2**).

Indoleamine 2,3-dioxygenase (IDO) is one of the three heme-containing dioxygenases [1]. This enzyme is involved in the kynurenine pathway which is the major pathway for the catabolism of the essential amino acid tryptophan (Trp), being responsible for catalysing the rate-limiting step of the Trp degradation to N-formylkynurenine [2]. Elevated Trp catabolism has been associated with rheumathoid arthritis [3] and cancer [4]. It has been shown that after chemotherapy, inhibition of IDO could delay the recurrence of tumour antigens tolerance [5], and also that its inhibition could be a new route to improve immunity to leishmania-infected humans [6]. As part of our research aimed at the synthesis of potential IDO inhibitors, using indole as a scaffold, 1-[(4-methylbenzene)sulfonyl]indole-3-carbaldehyde (**1**, Figure 1), was synthesized and its crystal structure determined.

In a previous contribution in this area [7], we reported the crystal structure of a closely related compound, 3-ethenyl-1-[(4-methylbenzene)sulfonyl]indole (**2**), where an ethenyl moiety was in the 3-postion rather than the carbaldehyde of compound **1** (Figure 1). It is noted that in both **1** and **2** there are two independent molecules in the asymmetric unit, *i.e.*, *Z*' = 2, and that in each case these are conformers, each being related to each other via a rotation about the central S-N bond. As this situation seemed unusual [8,9], a search of the Cambridge Structure Database (CSD) [10] was performed. For all-organic molecules only, there were 148,842 hits for structures crystallising with *Z*' = 1 compared with 20807 with *Z*' = 2, *i.e.*, indicating examples of structures *Z*' = 2 are only 14% as likely to ocurr, compared with structures with *Z*' = 1. With this background, a search was conducted for structures related to **1**, *i.e.*, 1-(arylsulfonyl)indole derivatives, to ascertain how prevalent this phenomenon was for this class of compound. The results of this survey are also presented herein. Finally, DFT calculations on **1**, **2** and related compounds were performed in order to understand the energetics of these systems, in particular the nature of the rotational barrier around the S-N bond.

## 2. Results and Discussion

**Figure 2:**
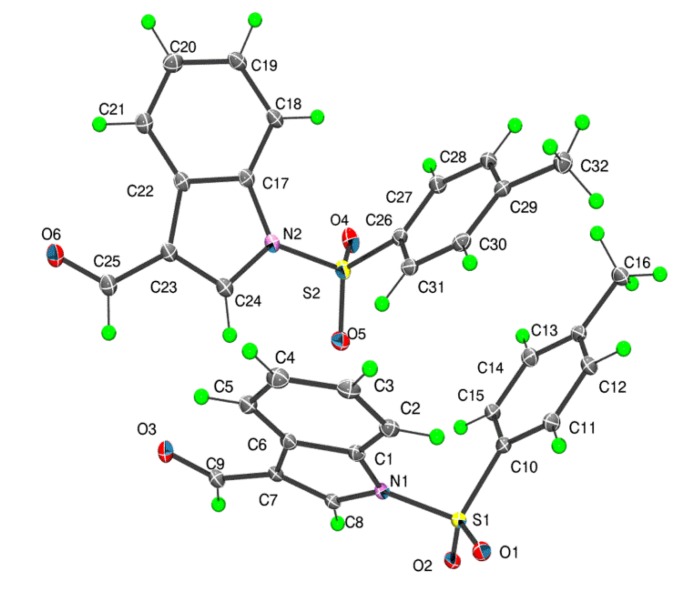
Molecular structures for the two independent molecules of **1** showing the atom numbering scheme.

There are two independent molecules in the asymmetric unit of **1** which are shown in Figure 2. The dihedral angles between the fused ring system (r.m.s. deviations = 0.011 and 0.022 Å for the N1- and N2-containing rings, respectively) and the benzene ring are close, *i.e.*, 79.08(6)° and 72.83(5)°, respectively. The values found in similar structures are 82.98(12)° and 84.46(13)° for the two independent molecules of 3-ethenyl-1-(4-methylphenylsulfonyl)-1*H*-indole (**2**) [7]; 80.37(8)° for (2-methyl-1-(phenylsulfonyl)-1*H*-indol-3-yl)methanol [11], 77.41(5)° for ethyl 2-bromo-3-(1-phenyl-sulfonyl-1*H*-indol-3-yl)acrylate [12] and 66.47(15)° for benzyl(3-bromo-1-(phenylsulfonyl)indol-2-ylmethyl)(*p*-tolyl)amine [13]. In **1**, each carbaldehyde moiety is almost co-planar with the ring to which it is connected as seen in the values of the C8-C7-C9-O3 and C24-C23-C25-O6 torsion angles of −175.4(2)° and −174.1(2)°, respectively. Further discussion on the relationship between the two independent molecules in **1** is found below in the context of the DFT calculations performed on these species.

**Figure 3:**
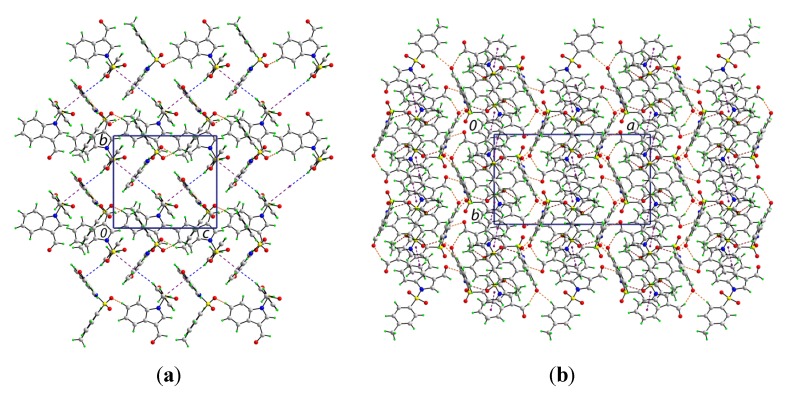
Views of the supramolecular association in the crystal structure of **1**: (**a**) supramolecular layer in the *bc*-plane, and (**b**) in projection down the c-axis showing the stacking of layers along the *a*-axis. The π···π, C-H···O (within layers), C-H···O (between layers), and C-H···π interactions (obscured in the (**b**) projection) are shown as purple, brown, orange and blue dashed lines, respectively.

The crystal structure features a range of intermolecular interactions as summarized in Table 1, with the most significant of these being π···π interactions occurring between the tolyl ring of the S1-containing molecule and the C_6_ ring of the indole fused ring system of the S2-containing molecule. The two-molecule aggregates thus formed are connected into a supramolecular chain along the c-axis via C-H···O interactions involving each of the sulfoxide-O atoms of the S2-containing molecule whereby the O4 and O5 atoms accept H atoms bound to indole- and methyl- residues derived from the S1- and S2-containing molecules, respectively. Links between these chains are of the type C-H···π and involve a tolyl-H atom of the S1-containing molecule interacting with the C_6_ ring of the indole fused ring system of the S2-containing molecule, resulting in the formation of supramolecular layers in the *bc*-plane, as shown in Figure 3a. Links between layers are exclusively of the type C-H···O and involve both aldehyde-O3 and O6 atoms as well as the sulfoxide-O2 atoms as acceptors, Figure 3b.

**Figure 4:**
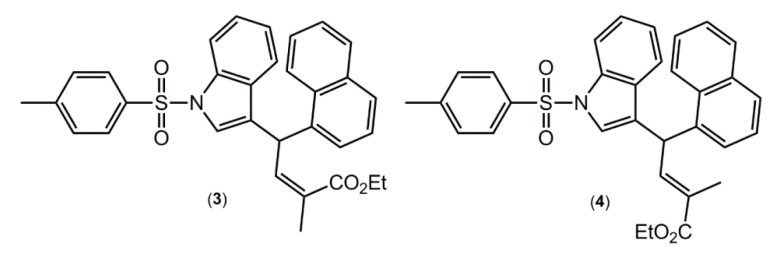
Chemical structures of ethyl 2-methyl-4-(1-((4-methylphenyl)sulfonyl)-1H-indol-3-yl)-4-(1-naphthyl)but-2-enoate (**3**), the *Z*-conformer with *Z*' = 1, and **4**, the *E*-conformer with *Z*' = 2.

A search of the CSD [10,14] resulted in 34 related 1-(arylsulfonyl)indole structures of which five have two independent molecules in the asymmetric unit, that is *Z*' = 2, a ratio consistent with the general findings mentioned in the Introduction (see the Supplementary Material of this article for the complete list of all 34 hits). Arguably, the most interesting of these structures are the (*E*)- and (*Z*)- conformers of ethyl 2-methyl-4-(1-((4-methylphenyl)sulfonyl)-1*H*-indol-3-yl)-4-(1-naphthyl)but-2-enoate [15] (Figure 4), where the (Z)- conformer crystallises with *Z*' = 1 (compound **3**), while the (*E*) conformer crystallises with *Z*' = 2 (compound **4**). These molecules were also subjected to DFT calculations along with **1** and **2**, as described below.

**Table 1:** Geometric parameters describing intermolecular interactions (A–H···B; Å) operating in the crystal structure of **1**.

A	H	B	A‒H	H···B	A···B	A‒H···B	Symmetry Operation
Cg(C10–C15)	-	Cg(C17–C22)	-	-	3.5917(13)	1.41(11)	2 – x, 1 – y, ½ + z
C3	H3	O4	0.95	2.56	3.370(3)	143	x, y, – 1 + z
C32	H32A	O5	0.98	2.55	3.401(3)	148	– x, 1 – y, – ½ + z
C31	H31	Cg(C1–C6)	0.95	2.99	3.901(2)	160	–x, 1 – y, ½ + z
C5	H5	O2	0.95	2.58	3.458(3)	155	½ – x, ½ + y, –½ + z
C8	H8	O6	0.95	2.40	3.129(3)	134	½ + x, ½ – y, z
C25	H25	O3	0.95	2.60	3.452(3)	150	–x, 1 – y, ½ + z
C27	H27	O3	0.95	2.33	3.175(3)	147	½ – x, –½ + y, ½ + z

**Figure 5:**
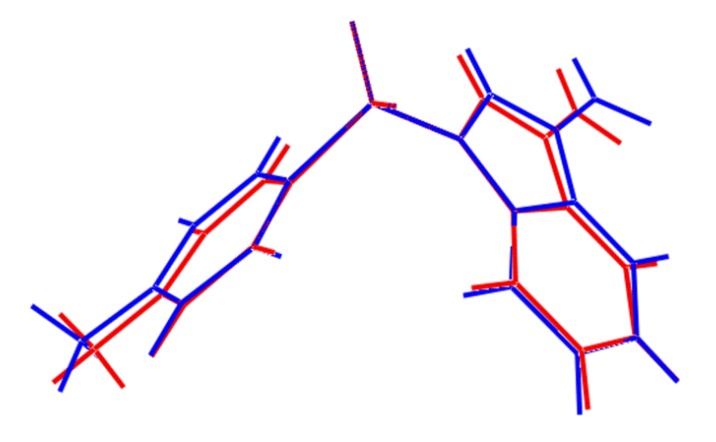
Superposition of the two independent molecules comprising the asymmetric unit of **1** [16], drawn so that the SO_2_ groups are superimposed. The inverted form of the S2-containing molecule (blue image) has been employed.

In all calculations the experimental structure, *i.e.*, as determined by X-ray crystallography, was the starting point for geometry optimisations. In cases where *Z*' = 2 and the independent molecules are conformers, as is the cases of **1** and **4** [15], calculations, as well as the Intrinsic Reaction Coordinate (IRC) calculations, were performed for both molecules. For **2** [7], where the two independent molecules were almost identical, the 180° related-conformer was generated. This was done so that comparable IRC calculations for **2** could be performed as for **1** and **4**. The two independent molecules of **1** are conformers, being twisted about the S-N and S-C bonds (Figure 2). At this point it is important to stress that the appearance of conformers is a solid-state effect. A variable temperature ^1^H-NMR study was conducted (see Supplementary Material) down to −50 °C in deuterated chloroform solution and no evidence was found for more than one conformation. As seen from the overlay diagram in Figure 5, where the S1-containing molecule has been super-imposed upon the inverted form of the S2-containing molecule using QMol [16], the molecular geometries closely resemble each other. The major difference in the molecules is quantified in the C10-S1-N1-C8 and C26-S2-N2-C24 torsion angles of 109.18(17)° and −116.34(18)°, respectively; a smaller difference in the O1-S1-C10-C11 and O4-S2-C26-C27 torsion angles of −2.1(2)° and 6.1(2)°, respectively, is noted.

**Figure 6:**
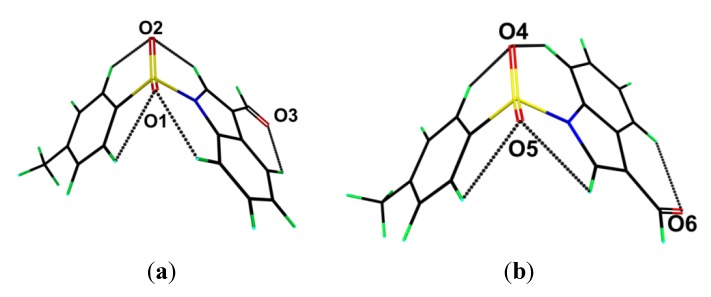
Geometry optimised molecules for the (**a**) S1- and (**b**) S2-containing molecules of **1**. Details of the intramolecular C-H···O interactions are tabulated in Table 2.

**Figure 7:**
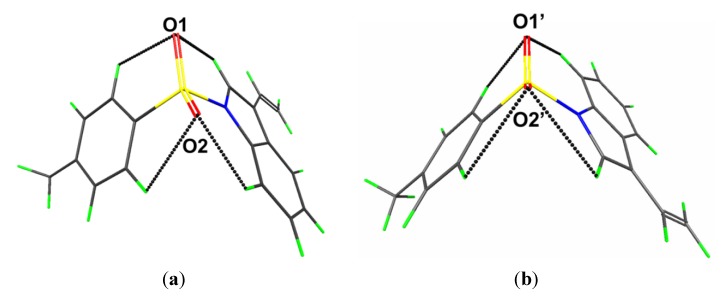
Geometry optimised molecules for the (**a**) S1- and (**b**) S1'-containing molecules of (**2**). Details of the intramolecular C-H···O interactions are tabulated in Table 2.

Geometry optimisation calculations conducted on the two independent molecules of each of **1** and **2** show that in each case both conformers optimise to the same energy-minimised molecule. This observation is readily ascribed to the fact that in both cases the structures are stabilized by comparable intramolecular C-H···O interactions as illustrated in Figure 6 and Figure 7 and quantified in Table 2, where the geometric parameters and NBO analyses of these interactions are listed.

**Table 2:** Geometric parameters describing intramolecular interactions (A–H···B; Å, °) for (**1**) and (**2**).

A	H	B	A–H	H···B	A···B	A‒H···B	NBO energy/kcal·mol^−1^
(**1**) S1-containing molecule							
C2	H2	O1	1.10	2.35	3.05	120.2	−2.09
C11	H11	O1	1.10	2.69	3.08	99.7	−0.29
C8	H8	O2	1.09	2.71	3.05	97.2	−0.26
C15	H15	O2	1.10	2.56	3.00	102.7	−0.60
C5	H5	O3	1.10	2.65	3.25	113.9	−0.76
(**1**) S2-containing molecule							
C18	H18	O4	1.10	2.36	3.05	120.0	−2.01
C27	H27	O4	1.10	2.70	3.08	99.5	−0.27
C24	H24	O5	1.09	2.70	3.04	97.4	−0.27
C31	H31	O5	1.10	2.56	3.00	102.8	−0.62
C21	H21	O6	1.10	2.65	3.25	113.9	−0.74
(**2**) S1-containing molecule							
C9	H9	O2	1.10	2.32	3.03	120.8	−2.32
C16	H16	O2	1.10	2.70	3.08	99.5	−0.27
C8	H8	O1	1.09	2.71	3.05	97.5	−0.25
C20	H20	O1	1.10	2.56	3.00	102.9	−064
(**2**) S1'-containing molecule							
C9	H9	O1'	1.10	2.33	3.04	120.6	−2.22
C20	H20	O1'	1.10	2.68	3.07	100.1	−0.31
C8	H8	O2'	1.09	2.70	3.05	97.7	−0.28
C16	H16	O2'	1.10	2.58	3.01	102.4	−0.57

NBO data calculated using the Giambiagi-Mayer and Wiberg models show that the S-O bonds have significant ionic character in both the energy minimised and in the transition state structures (Table 3), indicating that S^+^‒O^−^ is the best Lewis structure description, rather than S=O. The consequence of this is that each sulfoxide-O atom has three lone pairs. Moreover, the NBO results indicate that the strength of the S-N bond is due to n_N_→σ^*^_S-N_ and, and particularly, n_N_→σ^*^_S-C_ hyperconjugative interactions which do not persist in the transition state (Table 4). The S-N bond orders do not change significantly upon rotation as the n_O_→σ^*^_S-N _interactions do not achieve significant π-bond character from the n_N_→σ^*^_S-C_ and n_N_→σ^*^_S-O_ conjugation (Table 3 and Table 4), so that three-dimensional hyperconjugation results.

**Table 3:** Bond orders calculated for the optimised and transition state structures **1** and **2** ^a^.

Giambiagi-Mayer bond orders									
Bond	1–S1		1–S2	(1)TS1	(1)TS2	2–S1	2–S2	(2)TS1	(2)TS2
S-O	1.778		1.778	1.771	1.769	1.776	1.776	1.770	1.768
S-N	0.666		0.666	0.651	0.640	0.677	0.677	0.661	0.650
S-C	0.744		0.744	0.742	0.749	0.742	0.742	0.741	0.747
**Wiberg bond index**									
Bond	1–S1	1–S2	(1)TS1	(1)TS2	2–S1	2–S2	(2)TS1	(2)TS2	
S-O	1.484	1.484	1.484	1.482	1.483	1.483	1.482	1.481	
S-N	0.580	0.580	0.568	0.560	0.590	0.590	0.578	0.569	
S-C	0.694	0.694	0.693	0.701	0.691	0.691	0.691	0.698	

^a^ Bond orders are only listed for O1, as those for O2 have identical values.

**Table 4:** Stabilization energies (kcal·mol^−1^) of hyperconjugative interactions in the sulfonylgroup ^a,b^.

1				
Interaction	1–S1	1–S2	(1)TS1	(1)TS2
n_N_→σ^*^_S-C_	4.6	4.7	0.0	0.0
n_N_→σ^*^_S-N_	0.2	0.2	0.0	0.0
n_N_→σ^*^_S-O1_	2.0	1.8	6.0	5.9
n_N_→σ^*^_S-O2_	1.6	1.7	6.5	6.4
n_O1_ →σ^*^_S-C_	32.0	32.0	32.8	30.2
n_O1_ →σ^*^_S-N_	51.2	51.2	52.1	52.0
n_O1_→σ^*^_S-O1_	0.2	0.2	0.2	0.3
n_O1_ →σ^*^_S-O2_	30.3	30.3	30.3	30.3
**2**				
Interaction	2–S1	2-S2	(2)TS1	(2)TS2
n_N_→σ^*^_S-C_	4.5	4.5	0.0	0.0
n_N_→σ^*^_S-N_	0.2	0.2	0.0	0.0
n_N_→σ^*^_S-O1_	2.0	2.2	6.5	6.3
n_N_→σ^*^_S-O2_	2.0	1.8	6.5	6.0
n_O1_ →σ^*^_S-C_	32.2	32.2	33.0	32.6
n_O1_ →σ^*^_S-N_	50.1	50.1	51.1	51.6
n_O1_→σ^*^_S-O1_	0.2	0.2	0.2	0.2
n_O1_ →σ^*^_S-O2_	30.3	30.3	30.3	30.3

^a^ The oxygen lone pair interaction is the sum of interactions of the three lone pairs; ^b^ The listed energy values are those calculated for O1, as those for O2 have identical values.

**Figure 8:**
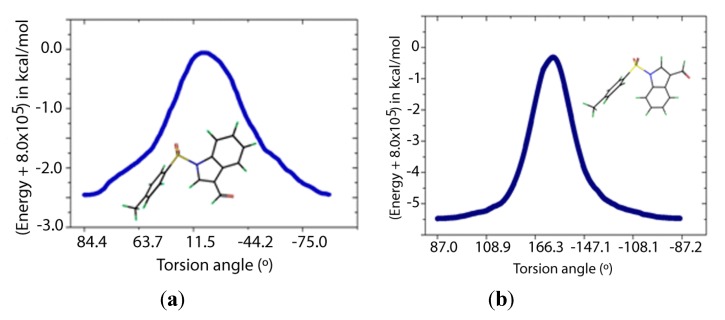
IRC of the rotational barrier about the S-N bond in **1**: (**a**) **1TS1 **and (**b**) **1TS2**. Insets are the corresponding molecular structures Torsion angle (°) (Energy + 8.0 × 10^5^) in kcal/mol.

**Figure 9:**
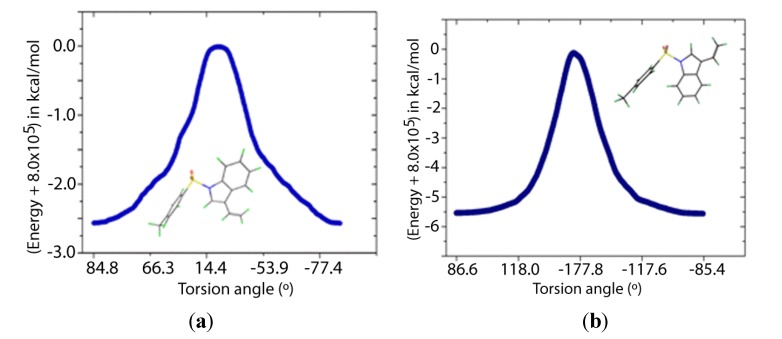
IRC of the rotational barrier about the S-N bond in **2**: (**a**) **2TS1 **and (**b**) **2TS2**. Insets are the corresponding molecular structures.

Two transition states for **1** and **2** were found (**TS1** and **TS2**, Figure 8 and Figure 9), with C-N-S-C torsion angles near 0° and 180° and energy barriers *ca* 2.5 and 5.5 kcal/mol, respectively (Table 5). The lone pairs of **TS1** have lower occupancies (Table 6) thus greater hyperconjugative effects than those of **TS2** according to the NBO analysis (Table 4) and that higher delocalization explains why the **TS1** has a more stable structure.

According to Table 7, the nitrogen and oxygen lone pairs in the transition states have higher repulsion than in the minimum energy structures. Therefore, the origin of the rotational barrier of these sulfonamides lies in the strong hyperconjugative effects of n_N_→σ^*^_S-C_ in the S-N bond coupled with the high repulsions between lone pairs in the transition state structures. This energy difference between the transition states affords an effective Gibbs free energy of activation close to that of TS1, which ensures that rotation occurs mainly via TS1. The relatively low rotational barrier indicates that there is a facile interconversion between the conformers.

**Table 5:** Relative energies (kcal·mol^−1^) and dihedral angles (°) of transition and ground states ^a^.

Structure	∆G^#^ (298.15 K)	∆E^#^_ZPE_	∆E^#^	∟C-S-N-C
**1**				
1	0.0	0.0	0.0	84.4
(1)TS1	3.8	2.3	2.4	2.0
(1)TS2	6.7	5.1	5.2	179.4
∆G*_eff_*^#^	3.8			
**2**				
2	0.0	0.0	0.0	84.8
(2)TS1	3.8	2.5	2.4	1.7
(2)TS2	7.0	5.3	5.4	179.5
∆G*_eff_*^#^	3.8			

^a^ ∆G***_eff_***^#^ = effective Gibbs free energy of activation.

As mentioned above, an interesting case is found in the structures of ethyl 2-methyl-4-(1-((4-methylphenyl)sulfonyl)-1*H*-indol-3-yl)-4-(1-naphthyl)but-2-enoate (Figure 4), for which the (*E*)-isomer **4** crystallizes with two independent molecules in the asymmetric unit, and the (*Z*)-isomer **3** with only one [15]; in the latter, the experimental structure was artificially manipulated to generate the other conformer. The conformers for each of **3** and **4** were also optimised. This showed that the conformers converged to the same energy in the gas-phase for **3** and **4**, respectively. Interestingly, the (*Z*)-isomer **3** has an energy 4.1 kcal·mol^−1^ lower than that calculated for the (*E*)-isomer **4**. An evaluation of the molecular structures, in particular the intramolecular C-H···O interactions, provides a clear explanation for the energy difference. Thus, the carbonyl-O atom in (3) forms two significant C‑H···O interactions (–11.9 and –14.6 kcal·mol^−1^) to provide considerable stability to the molecular structure. In **4**, analogous intramolecular C-H···O interactions are also present but these provide considerably less stabilisation to the molecular structure (−1.8 and −8.7 kcal·mol^−1^).

**Table 6:** Occupancies (***e***) of lone pairs in the transition states according to NBO analyses ^a–c^.

Orbital	(1)TS1	(1)TS2	Δn	(2)TS1	(2)TS2	Δn
n_N_	1.57042	1.57054	−0.000120	1.99357	1.99359	−0.00002
n_O1(1)_	1.99349	1.99354	−0.00005	1.67815	1.67935	−0.00120
n_O1(2)_	1.67855	1.67991	−0.00136	1.6318	1.63041	0.00139
n_O1(3)_	1.62852	1.62776	0.00076	1.99356	1.99358	−0.00002
n_O2(1)_	1.99350	1.99355	−0.00005	1.67799	1.67935	−0.00136
n_O2(2)_	1.67817	1.67999	−0.00182	1.63202	1.63044	0.00158
n_O2(3)_	1.62936	1.62766	0.00170	1.57986	1.58039	−0.00053
n_O3(1)_	1.99690	1.99679	0.00011			
n_O3(2)_	1.88105	1.88124	−0.00019			
Total	16.04996	16.05098	−0.00102	12.18695	12.18711	−0.00016

^a^ n_O3_ refers to the aldehyde-O3 atom which is found only in compound **1**; ^b^ numbers in parenthesis the label of the lone pair; ^c^ Δn = TS1 − TS2.

**Table 7:** Relative NBO Steric Exchange Energies between oxygen and nitrogen lone pairs.

Structure	∆E_steric_/kcal·mol^−1^		Structure	∆E_steric_/kcal·mol^−1^
1	0.0		2	0.0
(1)TS1	0.9		(2)TS1	0.9
(1)TS2	0.8		(2)TS2	0.9

## 3. Experimental

### 3.1. General Information

All reagents ware obtained from commercial sources. Melting points were determined on a Büchi B-545 (Büchi Labortechnik AG, Flawil, Switzerland); NMR data were obtained on a Bruker Avance DPX-300 spectrometer (Bruker BioSpin GmbH, Karlsruhe, Germany). 

### 3.2. Synthesis and Characterization

Into a previously flamed two-necked round-bottomed flask under a nitrogen atmosphere was poured the 1*H*-indole-3-carbaldehyde (0.145 g, 1 mmol, 1 eq.), CH_2_Cl_2 _(5 mL), TsCl (0.22 g, 1.15 mmol, 1.15 eq.), Et_3_N (0.20 mL, 1.5 mmol, 1.5 eq.) and 4-dimethylaminopyridine (4-DMAP, 0.012 g, 10 mol%, 0.1 eq.). This was followed by vigorous stirring for 2 h at room temperature. The mixture was acidified with a 1 N HCl solution and extracted with EtOAc, washed with a saturated solution of NH_4_Cl and H_2_O, and dried under MgSO_4_. The remaining solvent was removed under reduced pressure. The solid was washed with MeOH three times (yield = 88%). Crystals for X-ray analysis were obtained by slow evaporation from its EtOAc solution held at 293 K; M.p: 420–423 K. NMR ^1^H (CDCl_3_, 300 MHz) δ (ppm): 10.09 (s, 1H), 8,25 (dd, *J* = 9.2 and 0.7 Hz, 1H), 7,94 (dd, *J* = 9.2 and 0.7 Hz, 1H), 7.86 (t, *J* = 1.8 Hz 1H), 7.83 (t, *J* = 1.8 Hz 1H), 7.38 (qtd. *J*= 15.3, 8.0 and 1.5 Hz, 2H), 7.29 (d, *J* = 8.0 Hz, 2H), 7.25 (s, 1H), 2.37 (s, 3H). NMR ^13^C (CDCl_3_, 75 MHz) δ (ppm): 185.27, 146.15, 136.17, 135.30, 134.48, 130.33 (2C), 127.25 (2C), 126.35, 126.32, 125.07, 122.63, 122.44, 113.29, 21.68.

### 3.3. X-ray Data Collection and Processing

Data for a colourless block (0.20 × 0.25 × 0.30 mm) were collected at 100(2)K on an Agilent Super Nova-Dual diffractometer (Agilent Technologies Inc., Santa Clara, CA, USA) using Cu Kα radiation (mirror monochromator) and an Atlas detector using the ω scan technique to θ_max_ = 76.5°. No. of unique data = 5053, No. of parameters = 381, *R* (4882 data with *I* ≥ 2σ(*I*)) = 0.034, *wR*(all data) = 0.094. The structure was solved by direct methods [17] and refined by full-matrix least-squares on *F*^2^, with anisotropic displacement parameters for non-hydrogen atoms. The H atoms were geometrically placed (C—H = 0.95–0.98 Å) and refined as riding with *U*_iso_(H) = 1.2–1.5*U*_eq_(C). The weighting scheme used was *w* = 1/[σ^2^(*F*_o_^2^) + 0.065*P*^2^ + 0.201*P*] where *P* = (*F*_o_^2^ + 2*F*_c_^2^)/3) with SHELXL-97 [18] on *F*^2^. The programs WinGX [19] and ORTEP3 for Windows [19], PLATON [20], MarvinSketch 5.1.10 [21] and DIAMOND [22] were used for geometric calculations and to prepare crystallographic material for publication and depositing. Crystallographic data for the structural analysis have been deposited with the Cambridge Crystallographic Data Centre as CCDC 935801. Copies of this information may be obtained free of charge on application to CCDC, 12 Union Road, Cambridge CB2 1EZ, UK (fax: 44 1223 336 033; e-mail: deposit@ccdc.cam.ac.uk or www: http://www.ccdc.cam.ac.uk). 

### 3.4. Theoretical Calculations

All calculations were carried out using the PC GAMESS package with the B3LYP hybrid function, the STO-3G** basis set and wxMacMolPlt software for structure visualization [23,24,25,26,27,28,29,30]. The optimization algorithm was based on the Quadratic Approximation (QA) and the threshold gradient value used was 10^−5^ a.u. [31]. Frequency analyses were carried out to verify the nature of the minimum state of all the stationary points obtained. The Intrinsic Reaction Coordinate (IRC) calculations were done using the Gonzalez-Schlegel second-order method [32] with the former threshold gradient value and a step size between points of the reaction path of 0.2 a.u. The NBO donor-acceptor pairs were checked and second-order stabilization energies were calculated for the interaction studies [33,34,35,36,37,38]. The crystallographic structures were used as starting point for calculations.

### 3.5. CSD Survey Methodology

**Figure 10:**
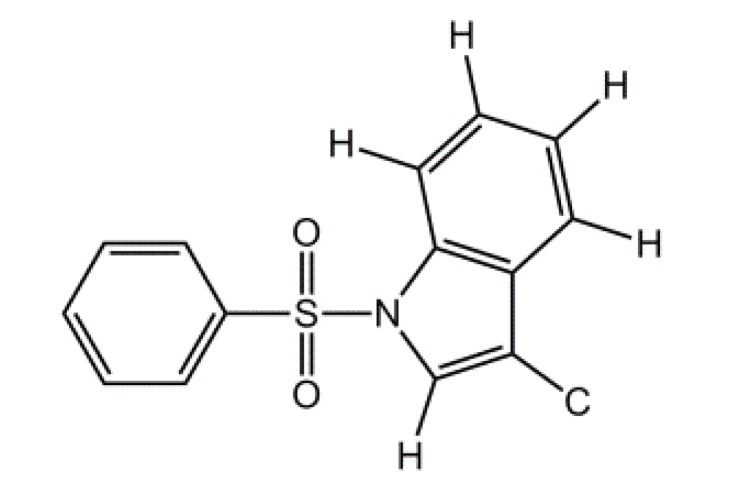
Generic 1-(arylsulfonyl)indole structure employed in the CSD search.

The Cambridge Structural Database (CSD: 5.32 + 4 updates) [10] was searched using CONQUEST (Version 1.14) [14] for the structural skeleton shown in Figure 10; structures featuring disorder or errors were excluded.

## 4. Conclusions

Two independent molecules comprise the asymmetric unit of 1-[(4-methylbenzene)sulfonyl]indole-3-carbaldehyde (**1**) which differ in terms of rotation about the central S-N bond; in deuterated chloroform solution only one conformation was found. DFT calculations show that the molecules converge to the same energy minimised gas-phase structure, and the IRC analysis shows the energy barrier to rotation about the S-N bond is *ca* 3.0 kcal·mol^−1^. Comparable energy barriers to rotation were found in related 1-(arylsulfonyl)indole derivatives. The NBO analysis showed that the n_N_→σ^*^_S-C_ hyperconjugative effects provide stabilization to the S-N bonding the ground state. While π-effects do not explain this rotational barrier, three-dimensional n→σ^*^_S-X _interactions and the increase the repulsion between lone pairs do.

## Supplementary Materials

Supplementary materials can be accessed at: http://www.mdpi.com/1420-3049/19/2/1990/s1.
