# Supplementary material for: Synthesis, Molecular and Crystal Structure Analysis of 1-(4-Methylbenzenesulfonyl)indole-3-carbaldehyde and DFT Investigation of Its Rotational Conformers
Source: Molecules. 2014 Feb 13;19(2):1990–2003. doi: 10.3390/molecules19021990 (PMC6270902; doi:10.3390/molecules19021990)

# Supplementary Materials

**Table S1.** Related 1-(arylsulfonyl)indole structures found in the Cambridge Structural Database.

| CSD code | Reference | Ref. No. |
|---|---|---|
| ABURUK | Lutz, G.; Pindur, U.; Schollmeyer, D. Asymmetrische Diels-Alder-Reaktionen an 2- und 3- Vinylindolen. PhD Thesis, University of Mainz, Mainz, Germany, 1994. | [1] |
| ABUSAR | Lutz, G.; Pindur, U.; Schollmeyer, D. Asymmetrische Diels-Alder-Reaktionen an 2- und 3- Vinylindolen. PhD Thesis, University of Mainz, Mainz, Germany, 1994. | [1] |
| ABUSOF | Lutz, G.; Pindur, U.; Schollmeyer, D. Asymmetrische Diels-Alder-Reaktionen an 2- und 3- Vinylindolen. PhD Thesis, University of Mainz, Mainz, Germany, 1994. | [1] |
| ATOYAJ | Caballero, E.; Alonso, D.; Pelaez, R.; Alvarez, C.; Puebla, P.; Sanz, F.; Medarde, M.; Tome, F. 1-Phthalimido-4-(3-indolyl)-2-siloxy-1,3-butadienes: Synthesis and Diels–Alder reactivity. *Tetrahedron Lett.* **2004**, *45*, 1631–1634. | [2] |
| BANREO | Sankaranarayanan, R.; Yogavel, M.; Velmurugan, D.; Sekar, K.; Babu, G.; Perumal, P.T.; Raj, S.S.S.; Fun, H.-K. 8-Chloro-4-[1-(phenylsulfonyl)indol-3-yl]-3a,4,5,9b-tetrahydro-3H-cyclopenta[c]quinoline. *Acta Crystallogr.* **2003**, *E59*, o49–o51. | [3] |
| BEHNAE | Kinsman, A.C.; Kerr, M.A. The Total Synthesis of (+) Hapalindole Q by an organomediated Diels Alder Reaction. *J. Am. Chem. Soc.* **2003**, *125*, 14120–14125. | [4] |
| BUJVUY | Jasinski, J.P.; Rinderspacher, A.; Gribble, G.W. Structures of three new (Phenylsulfonyl) indole derivatives. *J. Chem. Cryst.* **2010**, *40*, 40–47. | [5] |
| CIDBEX | Sonar, V.N.; Venkatraj, M.; Parkin, S.; Crooks, P.A. (*Z*)-2-[(1-Phenylsulfonyl-1H-indol-3-yl)methylene]-1-azabicyclo[2.2.2]octan-3-one semicarbazone. *Acta Crystallogr.* **2007**, *C63*, o277–o279. | [6] |
| FAKZOI | Mandal, D.; Yamaguchi, A.D.; Yamaguchi, J.; Itami, K. Synthesis of dragmacidin D via direct C-H couplings. *J. Am. Chem. Soc.* **2011**, *133*, 19660–19663. | [7] |
| FAKZUO | Mandal, D.; Yamaguchi, A.D.; Yamaguchi, J.; Itami, K. Synthesis of dragmacidin D via direct C-H couplings. *J. Am. Chem. Soc.* **2011**, *133*, 19660–19663. | [7] |
| HIZHEE | Zhu, J.; Zhang, X.Z.; Chen, S.Q.; Huang, X.H.; Zhang, Q.F. *J. Anhui Univ. Technol.* **2007**, *24*, 273. | [8] |
| IRIFAR | Zhao, F.; Fu, C.; Ma, S. Studies on the Intermolecular Hydroarylation of N-Ts- or N-Ac-Protected Indoles and 2,3-Allenoates. *Eur. J. Org. Chem.* **2011**, *2011*, 1227–1231. | [9] |
| IRIFEV | Zhao, F.; Fu, C.; Ma, S. Studies on the Intermolecular Hydroarylation of N-Ts- or N-Ac-Protected Indoles and 2,3-Allenoates. *Eur. J. Org. Chem.* **2011**, *2011*, 1227–1231. | [9] |
| IZEBEU | Sonar, V.N.; Parkin, S.; Crooks, P.A. (*Z*)-2-(1-Phenylsulfonyl-1H-indol-3-ylmethylene)-1-azabicyclo[2.2.2]octan-3-one and (*Z*)-(S)-2-(1-phenylsulfonyl-1H-indol-3-ylmethylene)-1-azabicyclo[2.2.2]octan-3-ol. *Acta Crystallogr.* **2004**, *C60*, o659–o661. | [10] |
| IZEBIY | Sonar, V.N.; Parkin, S.; Crooks, P.A. (*Z*)-2-(1-Phenylsulfonyl-1H-indol-3-ylmethylene)-1-azabicyclo[2.2.2]octan-3-one and (*Z*)-(S)-2-(1-phenylsulfonyl-1H-indol-3-ylmethylene)-1-azabicyclo[2.2.2]octan-3-ol. *Acta Crystallogr.* **2004**, *C60*, o659–o661. | [10] |
| IZUNAS | Meza-Leon, R.L.; Crich, D.; Bernes, S.; Quintero, L. Endo-selective quenching of hexahydropyrrolo[2,3-b]indole-based N-acyliminium ions. *J. Org. Chem.* **2004**, *69*, 3976–3978. | [11]. |
| IZUNIA | Meza-Leon, R.L.; Crich, D.; Bernes, S.; Quintero, L. Endo-selective quenching of hexahydropyrrolo[2,3-b]indole-based N-acyliminium ions. *J. Org. Chem.* **2004**, *69*, 3976–3978. | [11] |

**Table S1.** *Cont.*

| CSD code | Reference | Ref. No. |
|---|---|---|
| KEGCUV | Dockendorff, C.; Lautens, M.; Lough, A.J. 2-(1-Phenylsulfonyl-1H-indol-3-yl)-1,2-dihydronaphthalen-1-ol. *Acta Crystallogr.* **2006**, *E62*, o1030–o1032. | [12] |
| KERKIC | Kumar, G.S.; Chinnakali, K.; Balamurugan, R.; Mohanakrishnan, A.K.; Fun, H.-K. Ethyl 2-bromo-3-(1-phenylsulfonyl-1H-indol-3-yl)acrylate. *Acta Crystallogr.* **2006**, *E62*, o4972–o4974. | [13] |
| KIXTUG | Sankaranarayanan, R.; Velmurugan, D.; Raj, S.S.S.; Fun, H.-K.; Babu, G.; Perumal, P.T. 4-[1-(Phenylsulfonyl)indol-3-yl]-3a,4,5,9b-tetrahydro-3H-cyclopenta[c]quinolone. *Acta Crystallogr.* **2000**, *C56*, 475–476. | [14] |
| LAPBIP | Paramasivam, S.; Bhaskar, G.; Seshadri, P.R.; Perumal, P.T. (E)-3-Phenyl-2-(1-tosyl-1H-indol-3-ylcarbonyl)acrylonitrile. *Acta Crystallogr.* **2012**, *E68,* o683–o684. | [15] |
| LOXDOR | Jiang, B.; Yang, C.G.; Gu, X.H. A highly stereoselective synthesis of indolyl N-substituted glycines. *Tetrahedron Lett.* **2001**, *42*, 2545–2547. | [16] |
| LUDKUR | Mizoguchi, H.; Oguri, H.; Tsuge, K.; Oikawa, H. Divergent and expeditious access to fused skeletons inspired by indole alkaloids and transtaganolides. *Org. Lett.* **2009**, *11*, 3016–3019. | [17] |
| MORJUY | Jiang, B.; Yang, C.G.; Xiong, W.N.; Wang, J. Synthesis and cytotoxicity evaluation of novel indolylpyrimidines and indolylpyrazines as potential antitumor agents. *Bioorg. Med. Chem.* **2001**, *9*, 1149–1154. | [18] |
| PAWVIU | Zukerman-Schpector, J.; Wulf, G.D.; Stefani, H.A.; Vasconcelos, S.N.S.; Ng, S.W.; Tiekink, E.R.T. 3-Ethenyl-1-(4-methylphenylsulfonyl)-1H-indole. *Acta Crystallogr.* **2012**, *E68*, o1829–o1830. | [19] |
| QASXAK | Caballero, E.; Alonso, D.; Pelaez, R.; Alvarez, C.; Puebla, P.; Sanz, F.; Medarde, M.; Tome, F. Diels–alder reactivity of 4-aryl-1-phthalimido-2-siloxy-1,3-butadienes. *Tetrahedron* **2005**, *61*, 6871–6878. | [20] |
| QITMOV | Nieger, M. University of Bonn, Bonn, Germany, Personal communication, 2000. | [21] |
| ROCLIF | Forke, R.; Jager, A.; Knolker, H.-J. First total synthesis of clausine L and pityriazole, a metabolite of the human pathogenic yeast *Malassezia furfur*. *Org. Biomol. Chem.* **2008**, *6*, 2481–2483. | [22] |
| TEKTAE | Mohialdin-Khaffaf, S.; Persaud, K.C.; Pritchard, R.G. 3-Hexanoyl-1-tosylindole. A highly stereospecific preparation of 3-alkyl-substituted indoles. *Acta Crystallogr.* **1996**, *C52*, 2607–2609. | [23] |
| UBEBOU | Li, L.; Han, M.; Xiao, M.; Xie, Z. Proline-catalyzed enantioselective synthesis of aza-quaternary carbon derivatives. *Synlett* **2011**, *12*, 1727–1730. | [24] |
| VACKUH | Chakkaravarthi, G.; Panchatcharam, R.; Dhayalan, V.; Mohanakrishnan, A.K.; Manivannan, V. (Phenyl)(1-phenylsulfonyl-1H-indol-3-yl)methanone. *Acta Crystallogr.* **2010**, *E66*, o2895. | [25] |
| YEDYAI | Sonar, V.N.; Parkin, S.; Crooks, P.A. (Z)-2-[1-(4-Methylphenylsulfonyl)-1H-indol-3-ylmethylene]-1-azabicyclo[2.2.2]octan-3-one. *Acta Crystallogr.* **2006**, *E62*, o623–o625. | [26] |
| ZIKKAF | Schollmeyer, D.; Fischer, G.; Pindur, U. Dimeric 3-vinylindoles as potential antitumor active compounds: 1,1,3,4-Tetramethyl-3-(1-methyl-1H-indol-3-yl)-1,2,3,4-tetrahydrocyclopenta[b]indole and 1,1,3-Trimethyl-4-phenylsulfonyl-3-(1-phenylsulfonyl-1H-indol-3-yl)-1,2,3,4-tetrahydrocyclopenta[b]indole. *Acta Crystallogr.* **1995**, *C51*, 2572–2575. | [27] |
| ZILPAL | Vangveravong, S.; Nichols, D.E. Stereoselective synthesis of trans-2-(Indol-3-yl)cyclopropylamines: Rigid tryptamine analogs. *J. Org. Chem.* **1995**, *60*, 3409–3413. | [28] |

**Figure S1.** NMR acquisition parameters.

```
Current Data Parameters
NAME        Staley_090114
EXPNO                   2
PROCNO                  1

F2 - Acquisition Parameters
Date_            20140109
Time                16.14
INSTRUM             spect
PROBHD     5 mm TBI 1H/13
PULPROG              zg30
TD                  32768
SOLVENT             CDCl3
NS                     16
DS                      0
SWH              7878.151 Hz
FIDRES           0.240422 Hz
AQ              2.0797257 sec
RG                     57
DW                 63.467 use
DE                   6.50 use
TE                  272.9 K
D1             1.00000000 sec
TD0                     1

======== CHANNEL f1 =======
SFO1          600.1730171 MHz
NUC1                   1H
P1                   7.38 use
PLW1           9.00000000 W

F2 - Processing parameters
SI                  65536
SF            600.1700293 MHz
WDW                    EM
SSB        0
LB                   0.30 Hz
GB         0
PC                   1.00
```

**Figure S2.** NMR spectra at different temperatures.

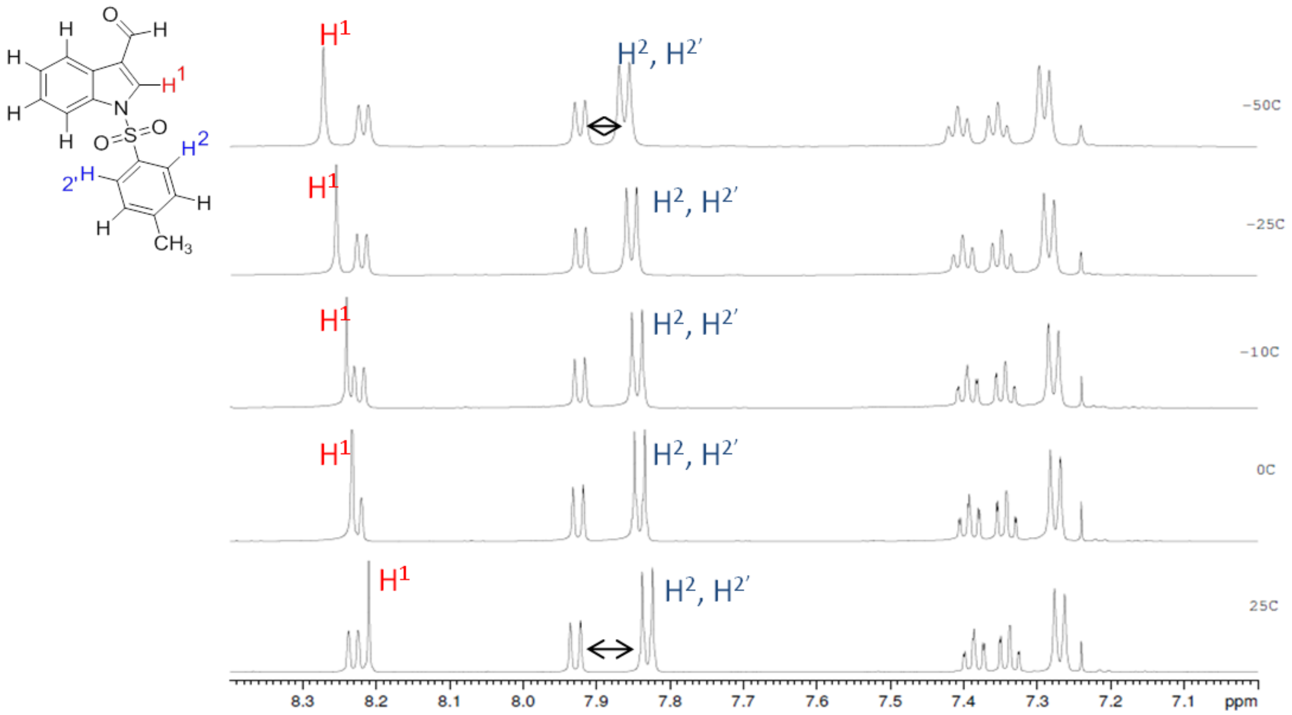

Supplement: Supplementary file 1 [file molecules-19-01990-s001.pdf]
